# Kelulut Honey Improves Folliculogenesis, Steroidogenic, and Aromatase Enzyme Profiles and Ovarian Histomorphology in Letrozole-Induced Polycystic Ovary Syndrome Rats

**DOI:** 10.3390/nu14204364

**Published:** 2022-10-18

**Authors:** Datu Agasi Mohd Kamal, Siti Fatimah Ibrahim, Azizah Ugusman, Siti Sarah Mohamad Zaid, Mohd Helmy Mokhtar

**Affiliations:** 1 Department of Physiology, Faculty of Medicine, Universiti Kebangsaan Malaysia, Kuala Lumpur 56000, Malaysia; 2 Department of Biomedical Sciences, Faculty of Medicine and Health Sciences, Universiti Malaysia Sabah, Kota Kinabalu 88400, Malaysia; 3 Department of Environment, Faculty of Forestry and Environment, Universiti Putra Malaysia, Serdang 43400, Malaysia

**Keywords:** Kelulut honey, folliculogenesis, steroidogenic, aromatase, PCOS

## Abstract

Polycystic ovary syndrome (PCOS) has been linked to aberrant folliculogenesis and abnormalities in the aromatase enzyme (Cyp19a1) and the steroidogenic enzyme, 17-alpha-hydroxylase (Cyp17a1) expression. It has been demonstrated that Kelulut honey (KH) improves both female and male reproductive system anomalies in animal studies. Here, we examined the effects of isolated and combined KH, metformin, and clomiphene in improving folliculogenesis, aromatase, and steroidogenic enzyme profiles and ovarian histomorphology in letrozole-induced PCOS rats. Letrozole (1 mg/kg/day) was administered to female Sprague–Dawley (SD) rats for 21 days to induce PCOS. PCOS rats were subsequently divided into six experimental groups: untreated, treatment with metformin (500 mg/kg/day), clomiphene (2 mg/kg/day), KH (1 g/kg/day), combined KH (1 g/kg/day) and metformin (500 mg/kg/day), and combined KH (1 g/kg/day) and clomiphene (2 mg/kg/day). All treatments were given orally for 35 days. We found that KH was comparable with clomiphene and metformin in improving the expression of Cyp17a1 and Cyp19a1, apart from enhancing folliculogenesis both histologically and through the expression of folliculogenesis-related genes. Besides, the combination of KH with clomiphene was the most effective treatment in improving the ovarian histomorphology of PCOS rats. The effectiveness of KH in restoring altered folliculogenesis, steroidogenic, and aromatase enzyme profiles in PCOS warrants a future clinical trial to validate its therapeutic effect clinically.

## 1. Introduction

Polycystic ovary syndrome (PCOS) is an interrelated disorder of the endocrine, metabolic, and reproductive systems [1]. It affects up to 20% of women globally, making it one of the most prevalent endocrine disorders among women of reproductive age, depending on the diagnostic criteria [2]. Women with PCOS may develop hyperandrogenism, anovulation, abnormal follicular development, hirsutism, and infertility [3]. PCOS is the leading cause of anovulatory infertility, with 40% of patients with PCOS experiencing infertility [1,3,4].

The pathophysiology and underlying aetiology of PCOS are still not well understood [5,6]. Among the postulated etiopathologies of PCOS are the aberrations in steroidogenesis [3,7], folliculogenesis [8], and aromatase enzyme action [9,10]. Genomic and molecular investigations have shown that increased androgen synthesis is an inherent steroidogenic abnormality in PCOS, particularly involving steroidogenic enzymes found in ovarian theca cells [11,12]. In theca cells, three enzymes are involved in various stages of the androgen synthesis process: 17-alpha-hydroxylase/17,20-lyase (Cyp17a1), 3-beta-hydroxysteroid dehydrogenase type II (HSD3B2), and cholesterol side-chain cleavage enzyme (Cyp11a1) [13]. In vitro studies using PCOS theca cells have shown that these enzymes are overexpressed, particularly Cyp17a1 [7,14].

Another dysregulated enzyme in PCOS is the aromatase enzyme encoded by cytochrome P450 family 19 subfamily A member 1 Cyp19a1 [9]. Aromatase catalyzes the rate-determining step during the conversion of androgens into oestrogens [15]. In women with PCOS, aromatase was found to be reduced [9,16]. Reduced aromatase enzyme activity causes hormonal imbalance, particularly hyperandrogenism [17,18].

In women with PCOS, hyperandrogenism causes folliculogenesis to be arrested [19]. Two phases of folliculogenesis in PCOS are reported to be arrested: arrested growth of antral follicles and arrested follicle development from the very early, gonadotropin-independent stages [20,21]. Folliculogenesis requires a group of specifically expressed genes to act as differentiation factors and biomarkers for folliculogenesis [22]. Among the genes are tumour necrosis factor alpha (*TNFα*), Kit ligand (*Kitlg*), basic fibroblast growth factor (*bFGF*), leukaemia inhibitory factor (*LIF*), bone morphogenetic protein-1 (*BMP-1*), and anti-Mullerian hormone (*AMH*) [22,23,24].

Currently, there is no definitive cure for PCOS. Therefore, PCOS treatment typically revolves around disease management and depends on the symptoms [25]. Currently, PCOS is commonly treated with two medications: metformin, which controls hyperglycaemia, and clomiphene citrate, which induces ovulation [26]. However, these medications are associated with several adverse effects. Metformin has been reported to have adverse gastrointestinal effects in women with PCOS [27]. Meanwhile, clomiphene has been linked to abdominal pain and uterine bleeding in patients with PCOS [28]. A systematic review by Sharpe et al. found that gastrointestinal side effects increased with a combined therapy of clomiphene and metformin [27]. Additionally, Moll et al. found that a higher percentage of women with PCOS who were treated with metformin discontinued the treatment due to the adverse effects [29]. A study in Australia discovered that 70% of women with PCOS used complementary medicine to manage their condition, with 12.2% of the participants reportedly experiencing adverse side effects [23]. Hence, finding a safe and evidence-based natural supplement such as honey is essential to meet the increasing demand.

Honey supplementation has been shown to be beneficial in female reproduction [30,31,32]. Kelulut honey (KH) is stingless bee honey with excellent antioxidative, anti-inflammatory, anti-cancer, and anti-diabetic properties [33]. It has been revealed that stingless bee honey, including KH, is predominantly composed of the unusual biologically active disaccharide trehalulose, a highly active antioxidant with a low glycaemic and insulinemic index [34,35]. Besides, a previous study demonstrated that KH contained phytochemical components such as 2,4-Di-tert-butyl phenol and octadecanoic acid, with antioxidant activity that could ameliorate the oxidative stress status of PCOS rats by improving catalase, superoxide dismutase, and glutathione peroxidase levels [36] In addition, KH is also found to be effective for reproductive health, as KH supplementation has been shown to improve the sperm quality of diabetic rats [37]. In another study, KH supplementation for pregnant women was reported to be safe and increase the haemoglobin level of pregnant women [38].

Previously, we have demonstrated that KH could normalise the disturbances in the hormones, oestrus cycle, and ovarian histomorphology in letrozole-induced PCOS rats [36,39]. With KH’s positive effect on the primary pathogenesis of PCOS, this present study examined the effects of isolated and combined KH, metformin, and clomiphene in improving folliculogenesis, aromatase, and steroidogenic enzyme profiles and ovarian histomorphology in letrozole-induced PCOS rats.

## 2. Materials and Methods

### 2.1. Honey Sample

KH was collected in April 2021 from a private bee farm in Negeri Sembilan, Malaysia. The herbal plants planted nearby were the source of nectar for the bees. The honey was maintained unprocessed and stored at 4 °C in glass bottles away from heat sources and direct sunlight.

### 2.2. Animal Preparation

All experimental procedures were reviewed and approved by the National University of Malaysia Animal Ethics Committee with the ethical approval code FISIO/FP/2020/MOHD HELMY/14-MAY/1104-JUNE-2020-MAY-2023. In this study, female Sprague-Dawley (SD) rats with body weights of between 120 and 150 g and with at least two continuous normal oestrus cycles were used. All experimental rats were received from the Laboratory Animal Research Unit (LARU), Faculty of Medicine, Universiti Kebangsaan Malaysia. All rats were housed under standard housing conditions (room temperature 24 ± 2 °C, 12 h light and 12 h dark cycle, one rat per cage) and were acclimatized for a week. All rats were fed a normal rat diet and provided with water ad libitum.

### 2.3. Animal Treatments

**Figure 1:**
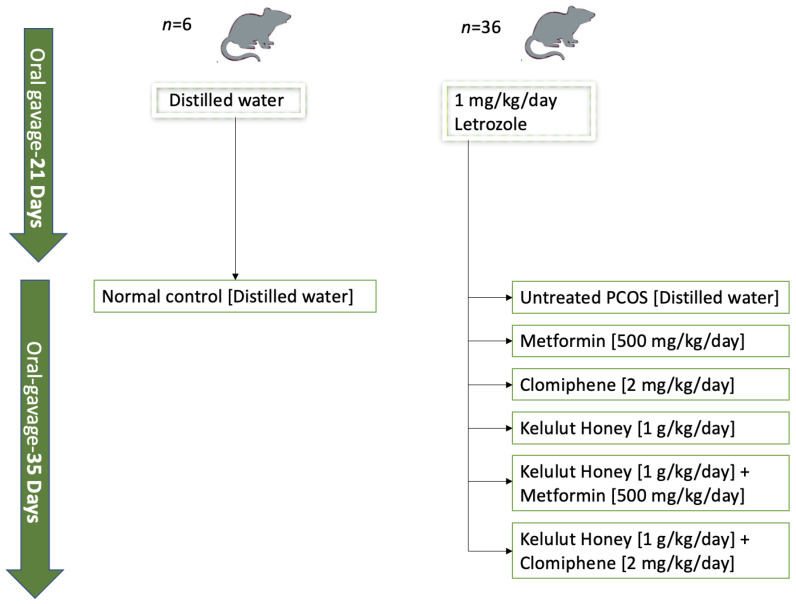
A flowchart illustrating the grouping and treatment of the animals.

The rats were randomly divided into two groups (Figure 1): the first group was the normal control group (*n* = 6), which consumed distilled water for the entire experiment (56 days), while the second group (*n* = 36) received letrozole orally once daily for 21 days at a dose of 1 mg/kg/day to induce PCOS [40]. PCOS was confirmed by examining the regularities of the oestrous cycle, blood glucose levels, and ovarian histomorphology [39].

The PCOS rats (*n* = 36) were subsequently divided into six treatment groups (*n* = 6 per group), as follows: untreated PCOS rats that were given distilled water, 500 mg/kg/day metformin, 2 mg/kg/day clomiphene, 1 g/kg/day KH, 1 g/kg/day KH with 500 mg/kg/day metformin, and 1 g/kg/day KH with 2 mg/kg/day clomiphene. All treatments were given via oral gavage for 35 days (Figure 1). The doses of metformin (500 mg/kg/day) and clomiphene (2 mg/kg/day) were chosen based on a previous study by Ndeingang et al. [41]. Meanwhile, the dose and duration of KH treatment (1 g/kg/day for 35 days) was determined based on our previous preliminary study [39]. Metformin and clomiphene are the drugs of choice for managing insulin resistance and ovulation failure in women with PCOS, respectively [26]. In this study, the groups receiving metformin and clomiphene were designed to evaluate the effect of KH treatment, while the combination of these drugs with KH was to discover the possible synergistic effect. The animals were euthanised by administering an overdose of ketamine and xylazine (0.3 mL/100 g body weight) [42].

### 2.4. Histomorphological Analysis of the Ovaries

Immediately after the rats were sacrificed, the right ovaries were taken, and the surrounding fat was removed before being preserved in a 10% neutral buffered formalin solution. Then, the ovaries underwent chemical processing and were paraffin-embedded. Next, ovarian tissues were sectioned into 5 μm thickness using a Leica RM2245 microtome (Leica Biosystems, Wetzlar, Germany), dried by air, dewaxed, sequentially rehydrated, and stained with haematoxylin and eosin solution. Afterwards, the slides were viewed using an Olympus BX40 light microscope (Olympus Corporation, Tokyo, Japan). Finally, the captured image was analysed with the ImageJ software.

The method used for counting the ovarian follicles was obtained from other studies [43,44]. Briefly, each serial segment of the ovary was examined to count the number of cysts, corpus luteum, atretic, and antral follicles. Healthy follicles were distinguished by the existence of an oocyte with a visible nucleus and a healthy granulosa cell layer. Antral follicles are described as follicles containing two or more layers of cuboidal granulosa cells. If the follicles have attenuated granulosa cells or pyknotic ovarian degeneration, they are classified as atretic. Meanwhile, the cystic follicle contained a fluid-filled cyst with an attenuated granulosa cell layer and thickened theca interna cell layer [43,44].

### 2.5. Protein Distribution Analysis by Immunohistochemistry (IHC)

The right ovary was harvested and blocked-in paraffin wax, as described earlier [42]. Ovarian tissues were then sectioned at 5 μm thickness. According to the manufacturing guidelines, the slides were immersed in an antigen retrieval solution (Dako, Glostrup, Denmark). Mouse and Rabbit Specific HRP/DAB IHC Detection Kit-Micro-polymer (Abcam, Cambridge, MA, USA) was used per the manufacturer’s guideline. Briefly, hydrogen peroxide was applied to the slides to inactivate the endogenous peroxidase, and the slides were then incubated with blocking serum. Next, the slides were treated with a rabbit polyclonal antibody against Cyp19a1 in a 1:200 dilution (Cat AB191093 Abcam) or a rabbit polyclonal antibody against Cyp17a1 in a 1:1000 dilution (Cat AB231794 Abcam). The slides were further incubated with an Abcam micro-polymer secondary antibody. The slides were incubated with the DAB substrate to visualise the protein expression. The slides were then dehydrated sequentially in alcohol and counterstained with haematoxylin solution. Finally, the slides were viewed using an Olympus BX40 light microscope (Olympus Corporation, Tokyo, Japan). The positive control tissue for Cyp19a1 is rat placental tissue, while Cyp17a1 is the rat testis and human adrenal tissues. Protein expression of Cyp19a1 and Cyp17a1 in the obtained images was analysed using ImageJ Fiji software (version 1.2; WS Rasband, National Institute of Health, Bethesda, Rockville, MD, USA) based on a validated protocol [45].

### 2.6. mRNA Expression Analysis by Real-Time Polymerase Chain Reaction (qPCR)

The left ovary was kept in RNA Later solution (Sigma Aldrich, Saint Louis, MO, USA) to stabilise and preserve the quality of the RNA in the tissue. Then, ovarian tissue lysates were powdered using a mortar and pestle in liquid nitrogen. The ovarian RNA was extracted using the Nucleospin RNA isolation kit (Macherey-Nagel, Duren, Germany) based on the producer’s guidelines. The RNA purity was evaluated by the 260/280 nm absorbance ratio (Gene Quant 1300, Cambridge, UK). Next, cDNA transcription was carried out using the OneScript^®^ Hot cDNA Synthesis Kit (Applied Biological Materials (ABM) Inc., Vancouver, BC, Canada). As a control, reverse transcriptase (RT)-free sample amplification was performed. The qPCR master mix was prepared using BlasTaq 2X qPCR MasterMix (Applied Biological Materials (ABM) Inc., Vancouver, BC, Canada). The reference gene used was glyceraldehyde-3-phosphate dehydrogenase (GAPDH).

Validated primers were obtained from Sigma-Aldrich (Saint Louis, MO, USA), as shown in Table 1. Real-time PCR was performed using the BioRad CFX96 Real-Time System. The following conditions were applied: The polymerase was activated for 3 min at 95 °C, then denaturation occurs for 15 s at 95 °C for 40 cycles, and then annealing and extension occur for 1 min at 60 °C for 40 cycles. The experiments were run in triplicates. Data were analysed using the comparative CT (2^−ΔΔCt^) method. The relative expression value of each amplicon was calculated by comparing the normalized value of each gene to the normalised value of the reference gene (GAPDH).

### 2.7. Statistical Analysis

All data were reported as a mean ± SEM. One-way ANOVA with post hoc Tukey’s multiple comparison tests were used to evaluate the differences among the groups using GraphPad Software (GraphPad Inc., San Diego, CA, USA). The statistical significance was set at *p* < 0.05. 

## 3. Results

### 3.1. Effects of KH on the Ovarian Histomorphological Changes

**Figure 2:**
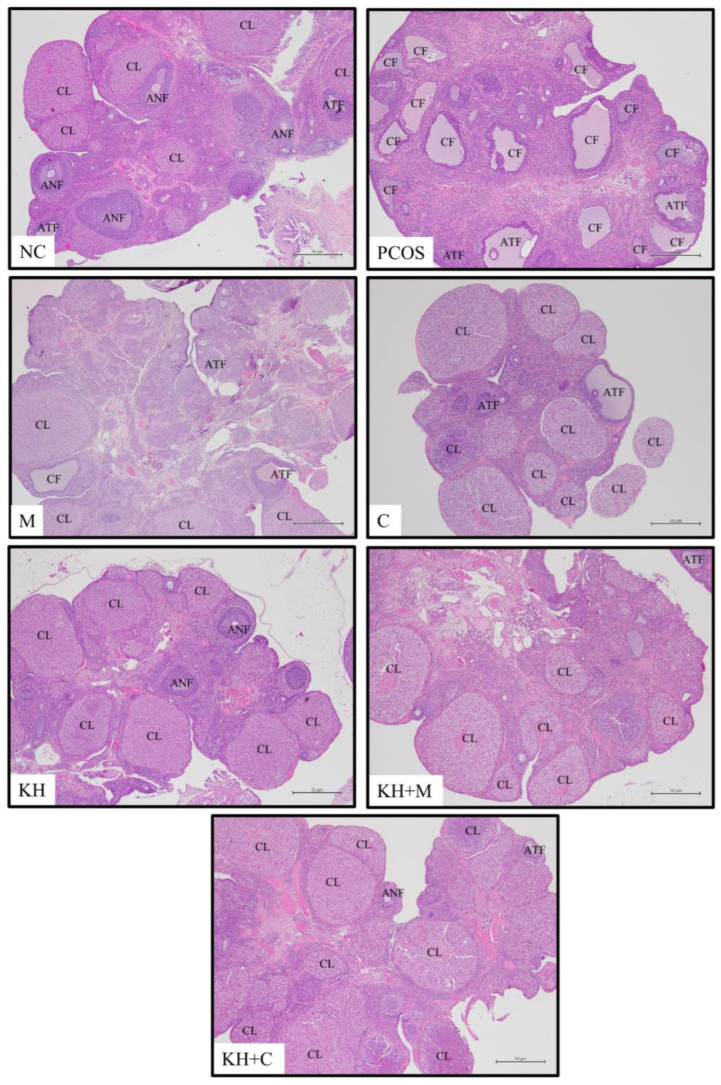
Effects of KH treatment on ovarian histomorphological changes. NC: normal control; PCOS: untreated PCOS; M: PCOS + Metformin; C: PCOS + Clomiphene; KH: PCOS + 1 g/kg/day of Kelulut honey; KH+M: PCOS + 1 g/kg/day of Kelulut honey + Metformin; KH+C: PCOS + 1 g/kg/day of Kelulut honey + Clomiphene; CL: corpus luteum; ANF: antral follicle; ATF: atretic follicle; CF: cystic follicle. Magnification 40×. Scale bar = 50 µm.

Figure 2 shows the effects of KH on the histomorphology of ovarian tissues. The normal control group ovaries displayed normal healthy histological appearances, including an abundance of well-formed corpus luteum and healthy follicles with fewer atretic and cystic. In contrast, the untreated PCOS group had more atretic and follicular ovarian cysts with fewer corpus luteum and antral follicles. Treatment with KH, metformin, clomiphene, or a combination of these treatments causes various alterations to the corpus luteum, antral, cystic, and atretic follicles that will be elaborated on in more detail.

**Figure 3:**
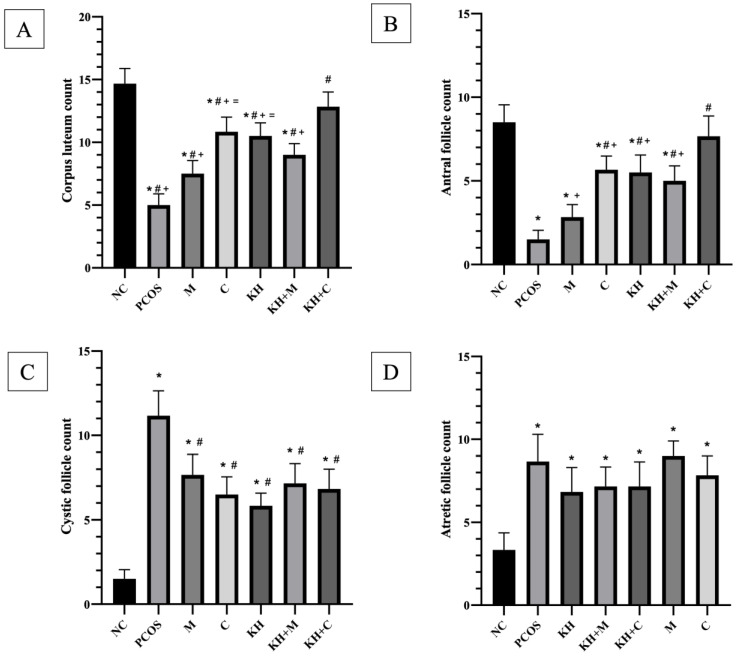
Effects of KH on (**A**) corpus luteum, (**B**) antral follicle, (**C**) cystic follicle, and (**D**) atretic follicle counts. NC: normal control; PCOS: untreated PCOS; M: PCOS + Metformin; C: PCOS + Clomiphene; KH: PCOS + 1 g/kg/day of Kelulut honey; KH+M: PCOS + 1 g/kg/day of Kelulut honey + Metformin; KH+C: PCOS + 1 g/kg/day of Kelulut honey + Clomiphene. * *p* < 0.05 significance against the normal control group, # *p* < 0.05 significance against the untreated PCOS group, + *p* < 0.05 significance against the KH+C group, = *p* < 0.05 significance against the M group *n* = 6 per treatment group.

Figure 3A illustrates the effect of KH on the corpus luteum count. Letrozole induction decreased the corpus luteum count in untreated PCOS rats compared with the normal control group (5.00 ± 0.36 vs. 14.67 ± 0.49, *p* < 0.05). A significant reversal of this reduction occurred (*p* < 0.05) by treatment with KH + clomiphene (12.83 ± 0.47), clomiphene alone (10.83 ± 0.48), KH alone (10.5 ± 0.43), KH + metformin (9.0 ± 0.37), and metformin (7.5 ± 0.43). Among the treatment groups, KH + clomiphene showed a significantly higher (*p* < 0.05) corpus luteum count than all other groups.

The antral follicle count is shown in Figure 3B. Antral follicles were decreased in untreated PCOS rats compared with the normal control group (1.5 ± 0.22 vs. 8.5 ± 0.42, *p* < 0.05). Treatment with combined KH + clomiphene (7.66 ± 0.49), clomiphene alone (5.66 ± 0.33), KH (5.5 ± 0.43), and combined KH + metformin (5.0 ± 0.36) significantly increased the antral follicle count (*p* < 0.05) compared to the untreated PCOS rats (1.5 ± 0.22). However, treatment with metformin alone (2.83 ± 0.31) did not change the antral follicle count compared to the untreated PCOS rats. As in the corpus luteum analysis, KH + clomiphene significantly increased (*p* < 0.05) the antral follicle count compared with the other groups.

Figure 3C demonstrates the effects of different treatments on the cystic follicle count. We found that the cystic follicle count was increased in the untreated PCOS rats compared with the normal control rats (11.17 ± 0.6 vs. 1.5 ± 0.22, *p* < 0.05). Treatment with KH (5.83 ± 0.3), clomiphene (6.5 ± 0.43), combined KH + clomiphene (6.83 ± 0.47), combined KH + metformin (7.2 ± 0.47), and metformin (7.67 ± 0.49) significantly reduced the cystic follicle count (*p* < 0.05) compared with the untreated PCOS rats (11.17 ± 0.6). No difference was recorded among the treatment groups.

The total atretic follicle count (Figure 3D) was shown to be significantly higher in the untreated PCOS rats (8.67 ± 0.66) and all the other groups in comparison with the normal control rats (3.33 ± 0.42). Nevertheless, no significant differences in the atretic follicle count were seen in the treatment groups compared with untreated PCOS rats.

### 3.2. Effects of KH on Folliculogenesis-Related Genes

**Figure 4:**
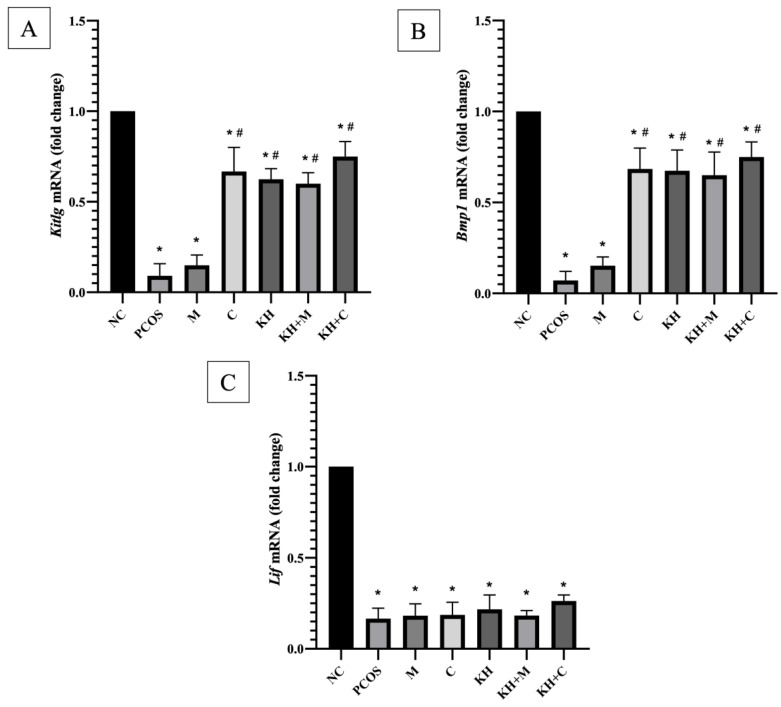
Effects of KH on (**A**) Kit ligand (kitlg) mRNA expression, (**B**) bone morphogenetic protein-1 (BMP1) mRNA expression and (**C**) leukaemia inhibitory factor (Lif) mRNA expression. NC: normal control; PCOS: untreated PCOS; M: PCOS + Metformin; C: PCOS + Clomiphene; KH: PCOS + 1 g/kg/day of Kelulut honey; KH+M: PCOS + 1 g/kg/day of Kelulut honey + Metformin; KH+C: PCOS + 1 g/kg/day of Kelulut honey + Clomiphene. * *p* < 0.05 significance against the normal control group, # *p* < 0.05 significance against the untreated PCOS group, *n* = 6 per treatment group.

Figure 4A demonstrates the effect of KH on *kitlg* mRNA expression. *Kitlg* mRNA expression was significantly downregulated in untreated PCOS rats compared with normal control rats (0.09 ± 0.03 vs. 1.00 ± 0.00, *p* < 0.05). The downregulation of the *kitlg* gene was significantly reversed (*p* < 0.05) by clomiphene treatment (0.67 ± 0.05), combined KH + clomiphene (0.75 ± 0.03), combined KH + metformin (0.59 ± 0.02), and KH (0.62 ± 0.02). However, there were no significant differences between the treatment groups. Additionally, the metformin-only group showed no significant difference compared with the untreated PCOS rats (0.15 ± 0.02).

As represented in Figure 4B, the mRNA expression of *BMP1* was also significantly downregulated in untreated PCOS rats compared with normal control rats (0.07 ± 0.02 vs. 1.00 ± 0.00, *p* < 0.05). Again, the downregulation of the *BMP1* gene was significantly reversed (*p* < 0.05) by clomiphene treatment (0.68 ± 0.04), combined KH + clomiphene (0.74 ± 0.03), combined KH + metformin (0.65 ± 0.05), and KH (0.67 ± 0.04). No significant differences were recorded among the treatment groups. Additionally, the metformin-only group showed no significant difference compared with the untreated PCOS rats (0.15 ± 0.01). The letrozole induction, as seen in Figure 4C, caused mRNA expression of *Lif* to be significantly downregulated in untreated PCOS rats compared with the normal control rats (0.17 ± 0.02 vs. 1.00 ± 0.00, *p* < 0.05). However, no significant differences were seen between all treatment groups compared to the untreated PCOS group.

### 3.3. Effects of KH on Cyp17a1 and Cyp19a1 mRNA Expression

**Figure 5:**
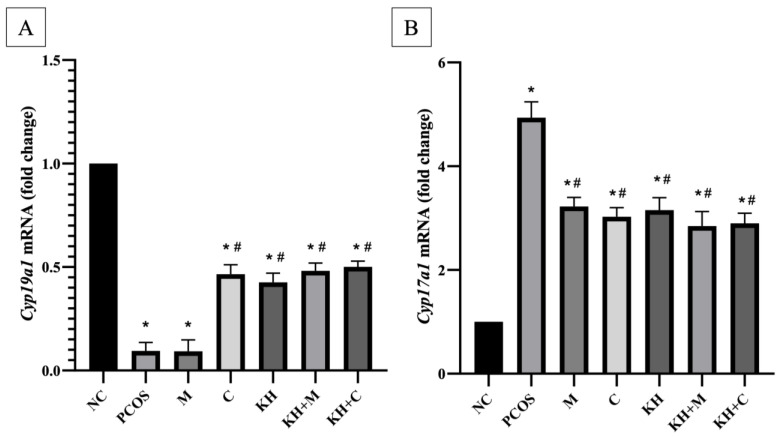
Effects of KH on (**A**) *Cyp19a1* mRNA expression (**B**) *Cyp17a1* mRNA expression. NC: normal control; PCOS: untreated PCOS; M: PCOS + Metformin; C: PCOS + Clomiphene; KH: PCOS + 1 g/kg/day of Kelulut honey; KH+M: PCOS + 1 g/kg/day of Kelulut honey + Metformin; KH+C: PCOS + 1 g/kg/day of Kelulut honey + Clomiphene. * *p* < 0.05 significance against the normal control group, # *p* < 0.05 significance against the untreated PCOS group. *n* = 6 per treatment group.

The effects of KH on *Cyp19a1* mRNA expression are shown in Figure 5A. Letrozole induction significantly downregulated the *Cyp19a1* mRNA expression in untreated PCOS rats compared with the normal control group (0.094 ± 0.01 vs. 1.00 ± 0, *p* < 0.05). Treatment with KH (0.42 ± 0.02), clomiphene (0.46 ± 0.02), combined KH + metformin (0.48 ± 0.01), and combined KH + clomiphene (0.5 ± 0.01) significantly increased the *Cyp19a1* mRNA expression (*p* < 0.05) compared to the untreated PCOS rats (0.094 ± 0.01). However, there were no significant differences noted between the treatment groups. Additionally, treatment with metformin alone (0.09 ± 0.02) did not change the *Cyp19a1* mRNA expression compared with the untreated PCOS rats.

Meanwhile, the *Cyp17a1* mRNA expression is shown in Figure 5B. Letrozole induction caused *Cyp17a1* mRNA expression to be upregulated significantly (*p* < 0.05) in untreated PCOS rats compared with normal control rats (4.93 ± 0.13 vs. 1.00 ± 0, *p* < 0.05). Treatment with combined KH + metformin (2.85 ± 0.12), KH + clomiphene (2.89 ± 0.08), clomiphene (3.03 ± 0.07), KH only (3.15 ± 0.11), and metformin (3.22 ± 0.08) significantly downregulated the *Cyp17a1* mRNA expression (*p* < 0.05) compared to the untreated PCOS group (4.93 ± 0.13). However, there were no significant differences seen between the treatment groups.

### 3.4. Effects of KH on Cyp17a1 and Cyp19a1 Protein Distribution

**Figure 6:**
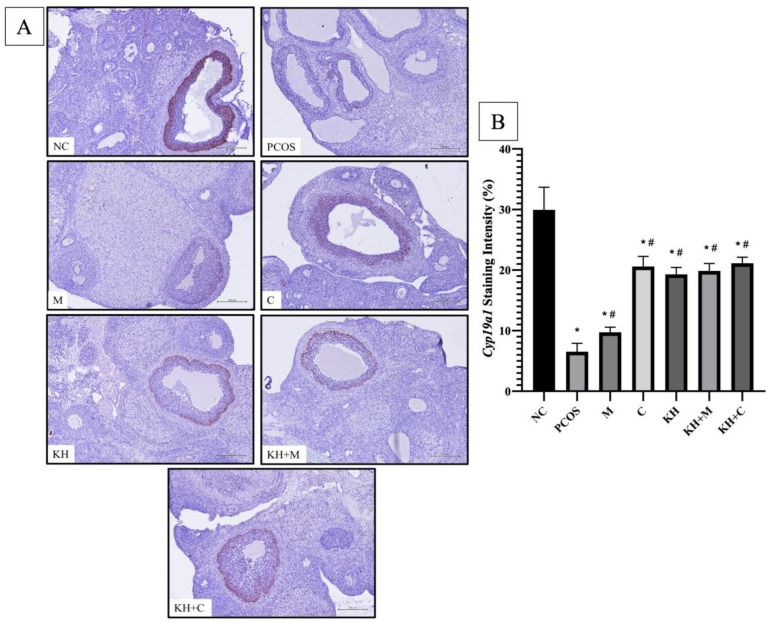
Effect of KH on Cyp19a1 (**A**) protein distribution (**B**) staining intensity. The dark brown denotes the Cyp19a1 antibody-binding site, which is present in the outer layer granulosa cells of antral follicles and preovulatory follicles. NC: normal control; PCOS: untreated PCOS; M: PCOS + Metformin; C: PCOS + Clomiphene; KH: PCOS + 1 g/kg/day of Kelulut honey; KH+M: PCOS + 1 g/kg/day of Kelulut honey + Metformin; KH+C: PCOS + 1 g/kg/day of Kelulut honey + Clomiphene. Scale bar = 100 μm. Magnification 100×. * *p* < 0.05 significance against the normal control group, # *p* < 0.05 significance against the untreated PCOS group. *n* = 6 per treatment group.

Figure 6A shows the distribution of the Cyp19a1 protein in rat ovaries. Positive DAB staining was localised at the outer granulosa cells of antral and preovulatory follicles. As seen in Figure 6B, staining intensity was significantly reduced in untreated PCOS rats compared to normal control rats (6.52 ± 0.56% vs. 29.94 ± 1.52%, *p* < 0.05). A significant reversal of this staining intensity reduction occurred (*p* < 0.05) by treatment with KH + clomiphene (21.13 ± 0.4%), clomiphene alone (20.59 ± 0.67%), KH alone (19.3 ± 0.46%), KH + metformin (19.85 ± 0.51%), and metformin (9.72 ± 0.34%). However, there were no significant differences between the treatment groups.

**Figure 7:**
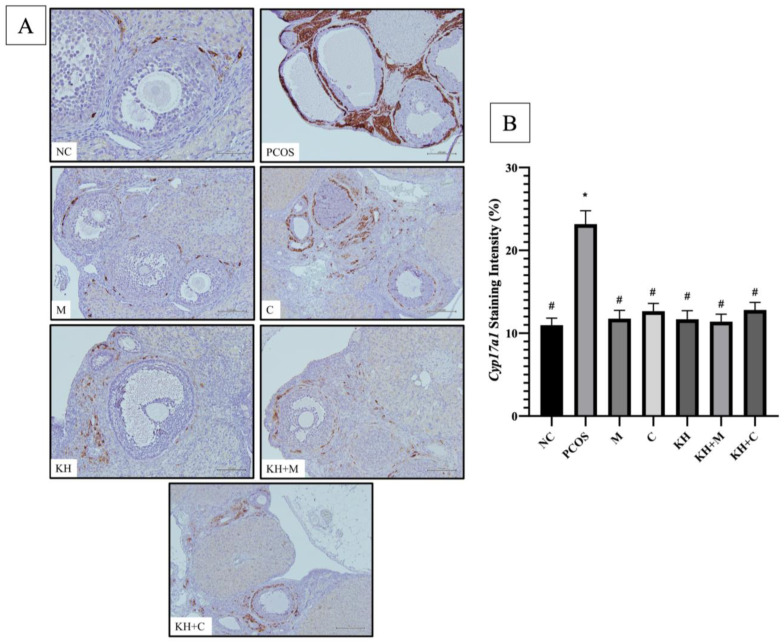
Effect of KH on Cyp17a1 (**A**) protein distribution (**B**) and staining intensity. The dark brown denotes the Cyp17a1 antibody-binding site, which is present in the theca cells of large antral and preovulatory follicles. NC: normal control; PCOS: untreated PCOS; M: PCOS + Metformin; C: PCOS + Clomiphene; KH: PCOS + 1 g/kg/day of Kelulut honey; KH+M: PCOS + 1 g/kg/day of Kelulut honey + Metformin; KH+C: PCOS + 1 g/kg/day of Kelulut honey + Clomiphene. Scale bar = 200 μm. Magnification 100×. * *p* < 0.05 significance against the normal control group, # *p* < 0.05 significance against the untreated PCOS group. *n* = 6 per treatment group.

Figure 7 shows the Cyp17a1 protein distribution. DAB staining was found in theca cells of large antral and preovulatory follicles. Figure 7B shows that the staining intensity of Cyp17a1 was significantly increased in untreated PCOS rats compared with normal control rats (23.17 ± 0.65% vs. 10.96 ± 0.35%, *p* < 0.05). The increase in staining was significantly reversed (*p* < 0.05) by treatment with KH + clomiphene (12.81 ± 0.37%), clomiphene alone (12.65 ± 0.38%), KH alone (11.68 ± 0.42%), KH + metformin (11.39 ± 0.37%), and metformin (11.76 ± 0.41%). However, no significant differences were seen between the treatment groups.

## 4. Discussion

Folliculogenesis can be divided into the early gonadotrophin-independent phase and the later gonadotrophin-dependent phase [24]. Bone morphogenetic proteins (BMP) play a crucial role in regulating the early phases of follicular growth. In early-stage follicles, the BMP system regulates gonadotropin receptors to induce gonadotropin [46]. In particular, BMP-1 regulates extracellular matrix formation during ovarian folliculogenesis [47]. Whereas leukaemia inhibitory factor (LIF) has been proven to stimulate the transition of primordial to primary follicle [48,49], modulate the differentiation of granulosa cells during pre-antral to antral follicle transition, and enhance oocyte meiotic competence [50]. The Kit ligand (KITL), with its receptor KIT, has various roles in follicle survival, oocyte development, and the stimulation of primordial follicles [51]. In a study using letrozole-induced PCOS rats, KITL and BMP were found to be downregulated in PCOS rats compared with normal rats. Similarly, our study found a significant downregulation in the expression of BMP-1 and KITL compared with normal control rats. Additionally, we recorded a downregulation of LIF expression in PCOS rats compared with normal control rats. KH treatment significantly reversed BMP-1 and KITL downregulation but did not affect LIF expression. Previously, *Allium fistulosum* treatment had also improved folliculogenesis by upregulating BMP-1 and KITL in PCOS rats [25].

In this investigation, clomiphene or KH combination with clomiphene or metformin significantly reversed the BMP-1 and KITL downregulation in PCOS rats. Clomiphene is known for facilitating folliculogenesis and, therefore, ovulation [52]. The binding of clomiphene to the hypothalamic oestrogen receptors stimulates the secretion of gonadotropins from the anterior pituitary, which eventually stimulates the recruitment of premature follicles to healthy antral follicles for ovulation [53]. Meanwhile, metformin is the drug of choice to manage diabetes in PCOS women. Apart from managing diabetes, metformin is also reported to be beneficial in ovulation. A study found that compared to the placebo, metformin improved the ovulation frequency in women with PCOS. However, metformin should not be used as the main drug for anovulation because clomiphene citrate or letrozole is more effective in improving ovulation and rates of live births in PCOS women [54]. Meanwhile, another study showed that metformin has limited action in improving reproductive disorders in women with PCOS [55]. In our study, metformin treatment alone did not affect the BMP-1, KITL, or LIF expression, which may be due to its modest effect on the ovulation rate, as mentioned above. Recently, a study found that treatments using medical-grade honey enhanced ovarian grafts’ viability by improving angiogenic and cell proliferation [56]. This affirms the potential of honey to improve ovarian physiology. However, further study on the effect of KH on LIF is needed to confirm its impact on this folliculogenesis factor.

The beneficial effect of KH on folliculogenesis is also reflected histologically. We found that KH treatment reverses the aberrant effect of letrozole on PCOS rats. KH groups showed a significantly higher count of corpus luteum and antral follicles than the PCOS group. The higher count of corpus luteum indicates an increase in ovulation rate, while the antral follicle shows that the normal folliculogenesis process is occurring. Cystic follicles were found to be lower in the KH-treated group compared to the PCOS group. However, KH did not alter the high level of atretic follicles in PCOS rats. These results further validate our preliminary study findings on KH’s beneficial effect on PCOS rats’ ovarian histology [39]. Previously, KH was reported to improve oxidative stress status and normalise the hormonal and oestrus cycle disturbances in PCOS-induced rats [36]. These hormonal and oestrus cycle corrections bring back physiological healthy folliculogenesis, which is manifested by improvement in folliculogenesis-related factors and histology findings in this study. Oxidative stress and its enzymatic markers play roles in regulating the folliculogenesis progress, oocyte development, and ovarian steroidogenesis [57,58,59]. These findings suggest an interplay between improved oxidative stress and folliculogenesis in this study. Furthermore, several studies have indicated that the phenolic contents of stingless bee honey, or KH, are responsible for its beneficial antioxidant effects [60,61]. The natural trehalulose enriched with the phytochemical component in KH may be the biologically active compound responsible for the benefit achieved in this study. Additionally, Tualang honey supplementation was also found to improve antral and atretic follicles in the ovary of cadmium and BPA-induced rats [62,63]. The findings on honey’s beneficial effects on the ovary strengthened its therapeutic potential for the female reproductive system.

KH’s effect on ovarian histology was comparable with that of clomiphene, whereby the clomiphene-only group also showed significant improvement in corpus luteum, antral follicle, and cystic follicle counts. Clomiphene’s effect on PCOS’s ovarian histology was in accordance with a previous study [64]. In this study, metformin treatment significantly reduced the cystic follicle count and increased the corpus luteum count. However, metformin treatment did not affect the antral follicles count in PCOS rats. Similar findings were observed in the past on the metformin effect on PCOS rat’s ovarian histology [65]. Interestingly, the combination of metformin with KH significantly increased the antral follicle and corpus luteum counts and reduced the cystic follicle count. Rudic et al. revealed that the combination of *Aronia melanocarpa* with metformin reduced the cystic follicle count and increased the corpus luteum count in PCOS rats [65]. Meanwhile, the combination of KH with clomiphene recorded the highest restoration of corpus luteum and antral follicles compared to all other treatment groups. The cystic follicle count was also significantly reduced in this group. This shows that the combination of KH and clomiphene has the best effect in inducing ovulation and improving folliculogenesis in PCOS rats.

In rodent ovaries, Cyp17a1 is localised in theca cells of large antral and preovulatory follicles [66]. Our study recorded a similar finding whereby the Cyp17a1 protein distribution was significantly higher in theca cells of the large antral and preovulatory follicles of PCOS rats compared with the normal control rats. Besides, the mRNA expression of Cyp17a1 in the PCOS group was significantly higher than in the normal control rats. This finding agreed with another study, which showed a similar up-regulation of gene expression and staining intensity of Cyp17a1 in PCOS rats [25]. This study indicates that KH treatment significantly reduced Cyp17a1 gene expression and staining intensity to levels comparable with clomiphene- and metformin-only treatments. Furthermore, it has been shown that LH hypersecretion is among the causes of Cyp17a1 overexpression [66]. Recently, KH has been proven to reduce the elevated LH level in PCOS rats [36], which might be related to the KH effect on Cyp17a1. Moreover, Tualang honey was reported to regulate the hypothalamic–pituitary–adrenal axis in ovariectomised rats [67]. In our study, a KH combination with either drug also significantly reduced Cyp17a1 gene expression and staining intensity. Previous reports have shown that metformin downregulated steroidogenesis-related enzymes, including Cyp17a1 [68,69]. On the other hand, clomiphene has been reported to downregulate Cyp11a1 and the steroidogenic acute regulatory protein (StAR) in PCOS-induced rats [64]. Meanwhile, another study reported that *Allium fistulosum* treatment of letrozole-induced PCOS rats does not correct the aberrant in Cyp17a1 caused by the letrozole induction [25].

In sexually mature animals, aromatase is reported to be present in the granulosa cell layer of large antral healthy follicles, preovulatory follicles, and corpus luteum [70,71]. Aromatase’s expression was highest in the mural granulosa cells at the periphery of the follicle than in the granulosa cells closer to the antral cavity. It was found that aromatase is absent in cumulus granulosa cells [70,72,73]. Herein, we demonstrated a very low aromatase expression in the outer layer granulosa cells of antral and preovulatory follicles. We also recorded significantly lower Cyp19a1 mRNA expression in PCOS rats. This finding was similar to a previous study whereby reduced aromatase expression was recorded in PCOS rats [74]. With KH treatment, aromatase was significantly restored to a comparable level with the clomiphene-only group. It has been reported that hyperandrogenism is the main factor that contributes to aromatase deficiency in women with PCOS [10]. Previously, KH was shown to reduce hyperandrogenism in PCOS rats [36] which might explain its role in restoring the aromatase expression in this study. However, the specific molecular pathway mediated by KH remains to be elucidated. Besides, metformin also has been reported to downregulate aromatase mRNA expression in granulosa luteal cells [75]. Meanwhile, clomiphene has been shown to restore aromatase levels in PCOS-induced mice [76]. Previously, Yulin mixture [76] and *Ecklonia cava* extract [74] have also been proven to restore aromatase levels in PCOS animal models.

## 5. Conclusions

In conclusion, our experimental results demonstrate that KH improves ovarian folliculogenesis and the steroidogenic and aromatase enzyme profiles in PCOS rats. These effects of KH are comparable with clomiphene and metformin treatment. Meanwhile, the combination of KH with clomiphene is the most effective in improving the ovarian histomorphology of PCOS rats. However, the specific molecular mechanism underlying the KH effects in this study remains to be elucidated. Moreover, the effectiveness of KH in restoring the altered folliculogenesis and the steroidogenic and aromatase enzyme profiles in patients with PCOS warrants a future clinical trial to validate its therapeutic effect clinically.

## Figures and Tables

**Table 1:** The sequence of primers used.

Gene	Forward (F) and Reverse (R) Primer Sequence
*Cyp17a1*	F→ATCCTGAGGTGAAGAAGAAG R→CAGTAAACTCTCCAATGCTG
*Cyp19a1*	F→CTAACATCATTCTGAACATCGG R→CTGAAAATACCTGTAGGGAAC
*KITLG*	F→GTGCTCTCTTCAACATTAGG R→CTTGACTGTTTCTTCTTCCAG
*LIF*	F→AAACTCAATGCGACTACAG R→AATGACTTGCTTGTATGTCC
*BMP1*	F→GGGGAGAAGATATTCTGAAC R→CCTCCACATAGTCATACCAG
*GAPDH*	F→CTCAATGGGAACTTAACAGG R→CTCTGTATAAGCAAGGATGC

## Data Availability

The data is contained within the article.
